# Phenytoin intoxication with no symptoms correlated with serum drug level: a case study

**DOI:** 10.11604/pamj.2015.22.297.7956

**Published:** 2015-11-24

**Authors:** Mucahit Avcil, Ali Duman, Kenan Ahmet Turkdogan, Mucahit Kapci, Ayhan Akoz, Selcuk Eren Canakci, Yunus Emre Ozluer

**Affiliations:** 1 Adnan Menderes University, Department of Emergency Medicine, Aydin, Turkey

**Keywords:** Phenytoin intoxication, emergency service, serum level

## Abstract

In high-dose intake of phenytoin, which is used frequently to treatepilepsy, nystagmus, diplopia, nausea-vomiting, lethargy, confusion, seizure, and coma can be observed. In recent studies on phenytoin intoxication, in which seizure and coma were observed in drug levels greater than 50 ug/mL. The serum phenytoin level of apatient, who consumed approximately 100 pcs of 100 mg phenytoin tablets in an effort to commit suicide, and who had no pathological finding in her neurologic examination, was 124 ug/mL. High drug level and the absence of toxic effect (or the absence of toxic effect correlated with the drug level) indicates that cytochrome P450 is functioning, but there can be a mutation in the MDR1 gene. In our case study, we report on phenytoin intoxication in a patient having a high level of phenytoin but no symptoms correlated with serum drug level, as supported by the findings in the literature.

## Introduction

Phenytoin has been used as an anticonvulsant since the late 1930s. It blocks voltage-dependent sodium channels, limiting the propagation of seizure discharges. This response is mediated by a use-dependent and voltage-dependent decrease in the rate of recovery of voltage-activated sodium channels from inactivation [1]. The multi-drug resistance (MDR) gene family is a sub-family of the ATP-binding-cassette (ABC) transporter super family. While MDR1 is the gene coding the glycoproteins that are responsible for drug transport, MDR2/3 are the genes coding the glycoproteins that do not play a role in drug transport [2]. Among the MDR1 gene products, phosphorylation of P-glycoprotein (PGP) regulates the transport of drugs out of cells [3]. PGP-expressing cells increase the concentration gradient of a drug [4]. This paper reports on phenytoin intoxication in a 36-year-old female patient having a serum phenytoin level of 124 ug/mL and no symptoms correlated with serum drug level, as supported by findings from the literature.

## Patient and observation

A 36-year-old female patient, who had a history of bipolar disorder, was brought to our emergency department by her relatives after taking 100 pcs of 100 mg tablets containing phenytoin with the intention of committing suicide. The vital signsof the patient at the time of admission were:blood pressure (BP): 100/60 mmHg, pulse:93 pulse/min, saturated O2: 100%, fever: 36.7°C, and respiratory rate: 22/min. The patient wasconfused and prone to sleeping, and all other system examinations were normal. Although the period of time that had elapsed from the intake of the druguntilthe administrationofemergency health procedures was not clear, according to the anamnesis taken from her relatives, it ranged from 30 and 60 minutes. The laboratory test results for the patient, were: pH: 7,37, pCO2: 43, pO2: 97, HCO2: 22, glucose:154, urea:8 creatinine:0.62, Na: 135, K:3.68, Cl:103, albumin:4.1, total bilirubin: 0.4, direct bilirubin: 0.17, AST:10, ALT:11, GGT:14, ALP:52, LDH:145, AMYLASE:23, LIPASE:7, Beta hCG < 1.20 Hb:11.1, Htc:35.5, leucocyte:7.17, platelet:243000 Pt:12.5sn, INR:0.89, APTT:21.7, ethanol: <10.0 mg/dl, phenytoin: 124.6 ug/mL. Stomach lavage was applied to the patient via an orogastric catheter, and then 1mg/kg activated charcoal was given. The patient, who was registered withthe national poison information system, was taken to our emergency intensive care unit for follow-up due to life threatening injury. After the initial dose of 1 mg/kg activated charcoal, additional doses of 0.5 mg/kg every 6 to 8 hours were administered to the patient for a total of 60 hours. Limitation of movement was prescribed for the patient, who had agitation and clouding of consciousness on the first day. Daily phenytoin dose follow-up was conducted (Table 1). The phenytoin dose that was 124.6 ug/mL on the first day was 107.4 ug/mL on the second day, 110.4 ug/mL on the third day,113.2 ug/mL on the fourth day, 98.6 ug/mL on the fifth day, 69.0 ug/mL on the sixth day, and 66.5 ug/mL on the seventh day.The patient's level of confusion was followed-up inthe intensive care unit for 3 days. She appeared to have recovered on the firstday, but her agitation regressed and her follow-up continued in theemergency department observation unit for 4 days. The patient, who developed no additional pathology and whose existing complaints regressed, was transferred in a good general condition and stable vital signs, to the psychiatry department on the seventh day.

**Table 1:** Daily phenytoin level and clinical observation of the patient

	1. Day Intensive care	2. Day Intensive care	3. Day Intensive care	4. Day Observation Unit	5. Day Observation Unit	6. Day Observation Unit	7. Day Observation Unit
Phenitoin level	124,6 ug/mL	107,4 ug/mL	110,4 ug/mL	113,2 ug/mL	98,6 ug/mL	69,0 ug/mL	66,5 ug/mL
Clinical Observation	nausea-vomiting, confusion, agitation	clear consciousness	clear consciousness	clear consciousness	clear consciousness	clear consciousness	clear consciousness

## Discussion

Antiepileptic drugs (AEDs) are a major treatment consideration for patients with epilepsy; therefore, the efficient control of seizures is the main concern when choosing the appropriate drug and dosage. A number of factors, including patient age and polypharmacy, increase the risk of side effects for AEDs [5]. Moreover, as has been reported in the literature, the results from this present study showed that there was no relationship between serum levels of AEDs and their side effects [6]. Phenytoin acts by inducing voltage-dependent and use-dependent blockage of the sodium channels. Phenytoin is one of a handful of drugs for which the kinetics change from the first order,"in which the extent of metabolism is directly correlated with the amount of available drug", to saturation at therapeutic doses. Accordingly, at a plasma concentration of around 15 ug per ml, a moderate increase in the drug dose can cause an unexpectedly large increasein the plasma concentration. A starting dose of 5 mg per kilogram of body weight increases the plasma concentrations; in most patients, the target range total is 20 ug per ml, but the concentrations are higher in some patients, resulting in neurotoxic effects [7]. In general, the dose can be increased by l00 mg if the plasma drug concentration is 8 ug per ml or less, but no more than 50 mg should be added if the plasma drug concentration is higher than 8 ug per ml. Some patients can benefit from plasma concentrations above 25 ug per ml without sideeffects. Phenytoin can cause a range of dose-related and idiosyncratic adverse effects. Reversible cosmetic changes (gum hypertrophy, acne, hirsutism, and facial coarsening), can be troublesome even though they tend to be mild. Neurotoxic symptoms (drowsiness, dysarthria, tremors, ataxia, and cognitive difficulties) become increasingly likely when the plasma drug concentration exceeds 20 ug per ml [6]. Even if the serum drug level is high, in some cases the absence of a clinical picture, which is observed in the literature [8] (Table 2), can be explained by genetic disorders at the receptor level between the drug metabolism and its activity [9]. It has been acknowledged that, PGP, a MDR1 gene product, is also found on the apical faces of normal cells in the large and small bowel columnar epithelium, the brush border of the renal tubuli, the biliary canaliculi of the hepatocyte, the pancreatic ductuli, the glandular epithelial cells of the endometrium in pregnant women, and also in the capillary endothelial cells of the blood-brain and blood-testicle barriers; moreover, other than the MDR phenotype, PGP is involved in various physiologic processes and augments its expression with appropriate stimulus.PGP performs the MDR function not by membrane permeability regulation but by acting as an ATP-dependent efflux pump from inside the cell to outside the cell [10]. Phosphorylation of PGP regulates the exocytosis mechanism of the drugs [3].

**Table 2:** Correlation of serum phenytoin level and clinical effects

µg/ml (mg/L)	Clinical Effects
<10	Non effects
10-20	Horizontal nistagmus
20-30	Spontan nistagmus
30-40	Tremor,diplopia, vertical nystagmus, ataxia, hyperreflexia
40-50	Lethargy, disorientation, confusion, hyperactivity
>50	Coma, seizures

## Conclusion

While the cytochrome P-450 gene plays an active role in drug metabolism, the MDR1 gene playsan active role in the efficiency of the drug at the receptor level. Whether or not this results in an increase in the serum drug level, we think that the personalization of the drug level correlated with this gene mutation and the clinical findings are suitable after observing depletion of MDR1 and cytochrome P-450 genes in diseases requiring chronic drug use. In our case study, we considered that the absence of symptoms correlated with serum drug level could be caused by the deletions in the MDR1 gene or the cytochrome P450 gene.
